# Obstructive Jaundice as an Uncommon Manifestation of Metastatic Breast Cancer

**DOI:** 10.14740/wjon762w

**Published:** 2015-02-14

**Authors:** Ivan Budimir, Mateja Sabol Pusic, Marko Nikolic, Zdravko Dorosulic, Neven Ljubicic, Emil Stajduhar, Ivana Mise, Ljubica Vazdar, Bozena Sarcevic

**Affiliations:** a Department of Gastroenterology and Hepatology, Interventional Gastroenterology Unit, University Hospital Center “Sestre Milosrdnice”, Vinogradska Street 29, Zagreb 10000, Croatia; b Department of Internal Medicine, Cantonal hospital Cakovec, I. G. Kovacica 1e, Cakovec 40000, Croatia; c Department of Radiology, University Hospital Center “Sestre Milosrdnice”, Vinogradska Street 29, Zagreb 10000, Croatia; d Department of Clinical Citology, University Hospital Center “Sestre Milosrdnice”, Vinogradska Street 29, Zagreb 10000, Croatia; e Department of Oncology, University Hospital Center “Sestre Milosrdnice”, Ilica 197, Zagreb 10000, Croatia

**Keywords:** Bile duct obstruction, Breast cancer, Jaundice, Metastasis, Stent

## Abstract

Invasive ductal carcinoma is the most common type of breast cancer and accounts for about 70-85% of all invasive breast carcinomas. It primarily metastasizes to the bone, lungs, regional lymph nodes, liver and brain. Most of breast cancer recurrence occurs within the first 5 years of diagnosis, particularly for ER negative disease. Gastrointestinal tract involvement is very rare and is detected in only 10% of all the cases, and it usually derives from lobular breast cancer rather than the much more common cell type of ductal breast cancer. Early diagnosis is very important because it enables prompt and adequate choice of treatment and improves patient’s long-term prognosis. In this report we describe an unusual case of obstructive jaundice caused by metastases from invasive ductal breast cancer to the lymph nodes of the hepatoduodenal ligament with extramural compression of the distal common bile duct and tumor invasion to the lumen of the duct. Our goal is to emphasize possible diagnostic pitfalls and increase the clinical awareness and the importance of intensive follow-up in patients with breast cancer, even years after the initial diagnosis.

## Introduction

Breast cancer is with worldwide over a million newly diagnosed cases each year, the most common malignancy in women and one of the leading causes of cancer death among them [1]. The histological types of breast cancer which represent the majority of cases are infiltrating ductal carcinoma, infiltrating lobular carcinoma and mixed carcinoma, while other types such as metaplastic, mucinous, tubular, medullary and papillary carcinomas together account for less than 5% of invasive cancers. At the time of the diagnosis breast cancer can, by the extent of the disease, be stratified into three groups: early stage breast cancer, locally advanced disease and metastatic disease. The most common sites of breast cancer metastases are the bone, lung, liver and brain. Lymph node metastasis into the sentinel node and few surrounding nodes is regarded as a treatable local event and not metastatic breast cancer when occurring at primary presentation or later. Treatment options depend on many factors and require multidisciplinary approach, which has, according to the literature, been associated with reduction in mortality [2].

To the best of our knowledge and review of the literature there has been only few similar reports [3-5], but this is the only case presenting advanced breast cancer first manifested by isolated intra-abdominal lymph node metastases diagnosed by endoscopic retrograde cholangiopancreatography (ERCP) and treated by endoscopic stenting. With this paper we want to emphasize that diagnosis can be difficult and controversial when relapse occurs at uncommon sites, but quick and accurate diagnosis is needed for adequate treatment choice.

## Case Report

A 49-year-old female patient was admitted to the Gastroenterology Unit of our clinic in March 2013 because of pruritus and biochemical parameters of cholestasis. From her medical history we learned that in 2007 she was diagnosed having invasive ductal carcinoma (grade II, ER 70%, PR 15% and HER-2 negative). At her course of treatment, first she underwent left breast segmentectomy with axillary dissection, and after operative treatment she received six cycles of chemotherapy (AC protocol: doxorubicin plus cyclophosphamide), irradiation and hormonal therapy (tamoxifen). On her regular follow-ups at the oncologist there was no sign of disease recurrence until 2013.

**Figure 1:**
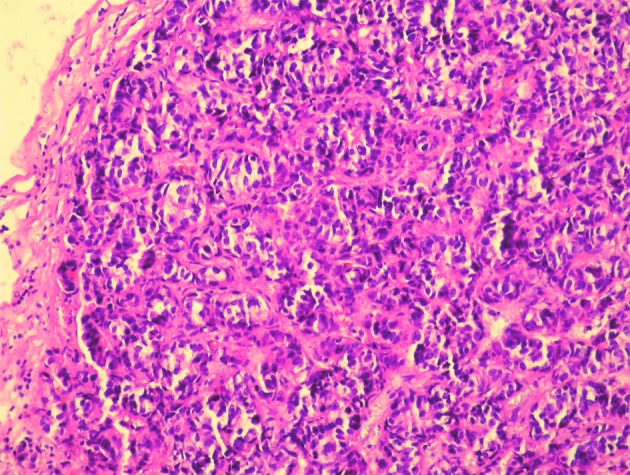
Histological analysis of the extirpated lymph nodes. Hemalaun-eosin stain showing infiltration with tubular formations and clusters of atypical epithelial cells which infiltrated the lymph node’s capsule (HE, × 20).

**Figure 2:**
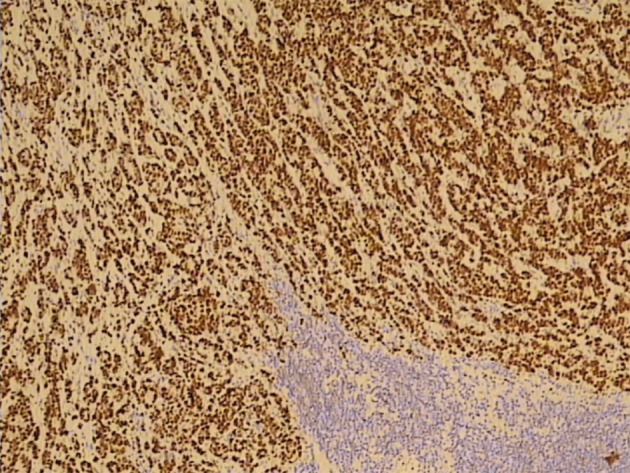
A strong positive nuclear reaction to estrogen receptor in 100% of tumor cells (IMH, × 10).

**Figure 3:**
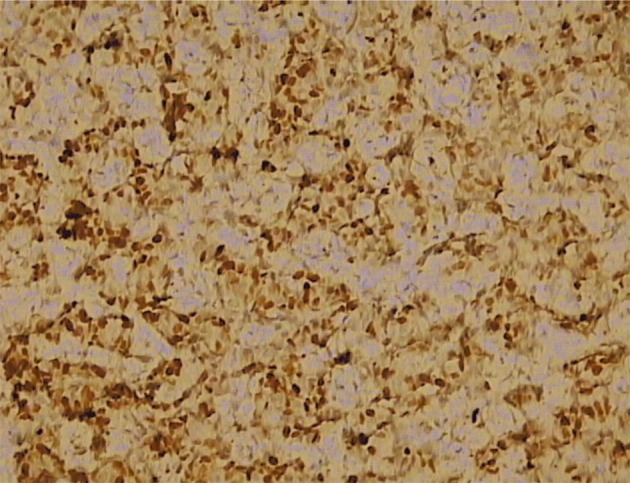
Immunohistochemically, moderate-to-strong nuclear reaction to progesterone receptor in 60% of tumor cells (IMH, × 20).

Three months before the admission to our clinic the patient underwent cholecystectomy with the extirpation of two lymph nodes of the hepatoduodenal ligament. Histological analysis of the extirpated lymph nodes showed infiltration with tubular formations and clusters of atypical epithelial cells which infiltrated the lymph node’s capsule (Fig. 1). Immunohistochemically, tumors cells showed a strong positive nuclear reaction to estrogen receptor in 100% of tumor cells (Fig. 2) and a moderate-to-strong reaction to progesterone receptor in 60% of tumor cells (Fig. 3). HER-2 receptor was negative, and proliferation index Ki67 was 60%. These results proved metastases of breast cancer cells into the extirpated lymph nodes.

In laboratory tests at the time of admission to our ward, we found slightly increased sedimentation rate (40) and elevated liver tests: AST 87 U/L, ALT 149 U/L, AF 366 U/L, GGT 146 U/L and bilirubin 33.4 µmol/L.

Abdominal ultrasound showed dilatation of the common hepatic duct (14 mm wide) and intrahepatic bile ducts and also enlarged para-aortal lymph nodes. PET CT scan showed pathological activity in left supraclavicular, retropectoral and retrosternal lymph nodes, infrahepatal lymph nodes and in the area of head, body and tale of pancreas.

**Figure 4:**
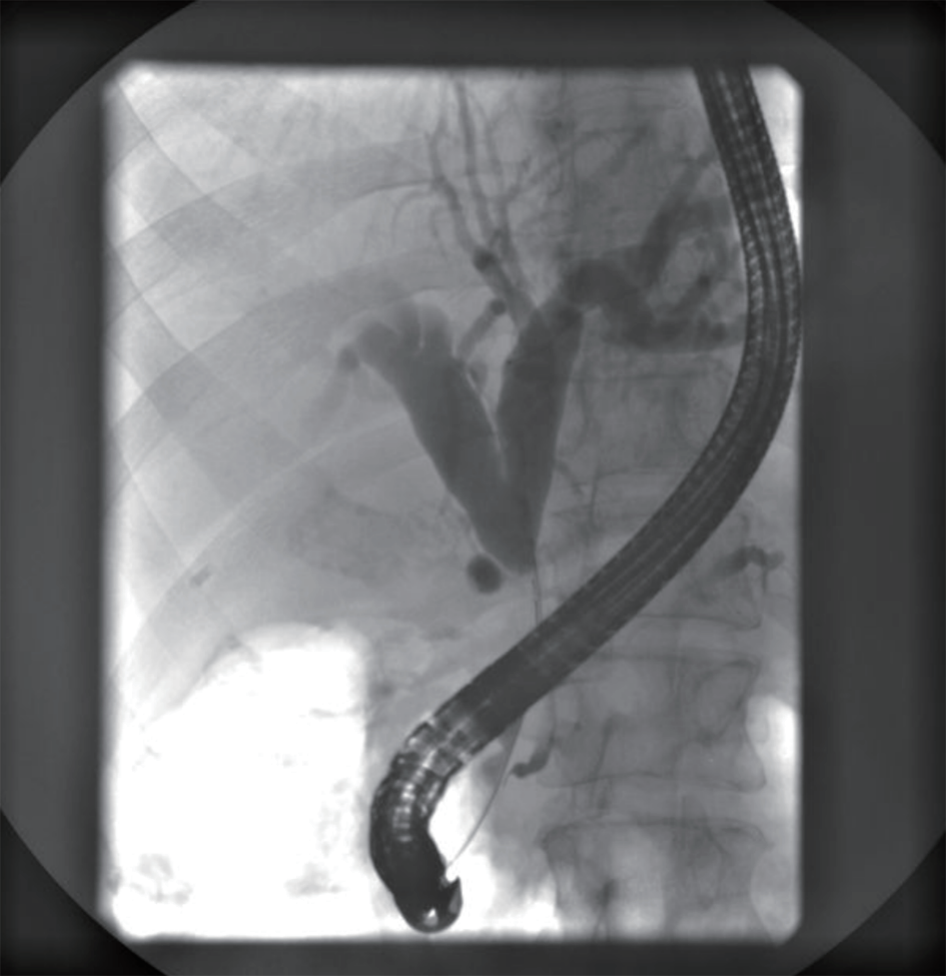
ERCP showing a subtotal stenosis of the common bile duct 3 cm in length with expressed prestenotic dilatation of both hepatic ducts and intrahepatic bile ducts.

**Figure 5:**
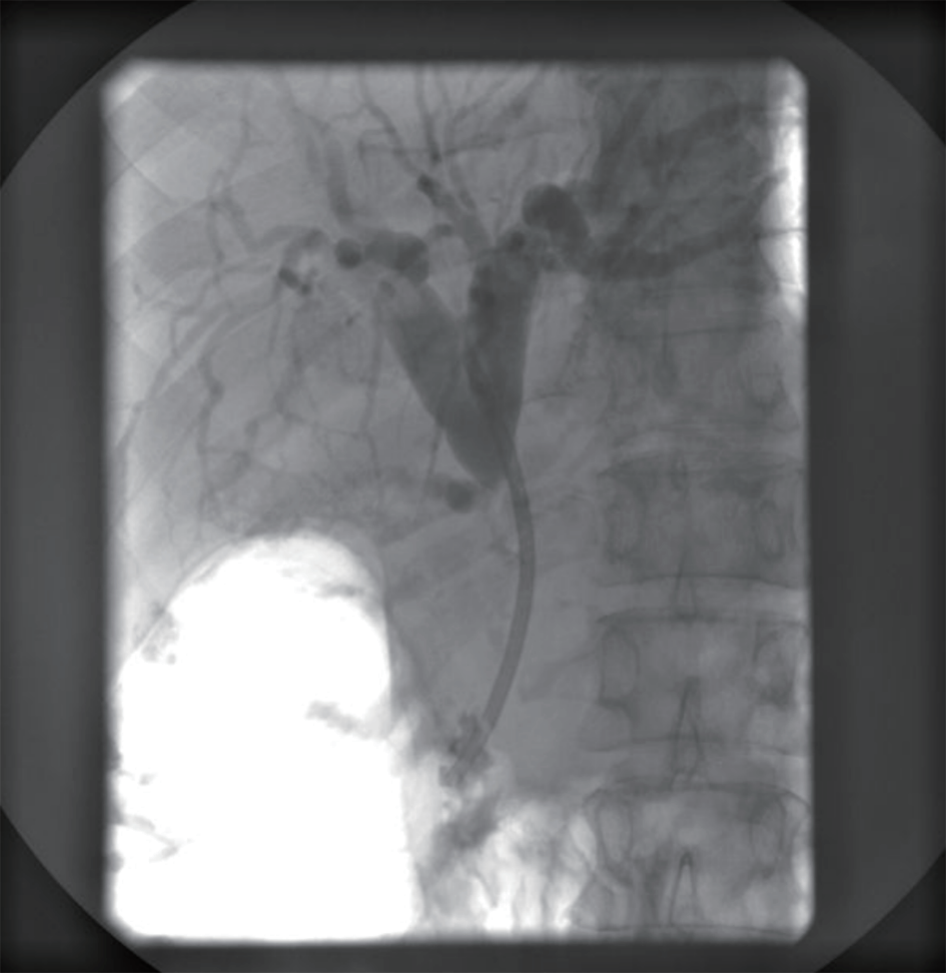
ERCP showing an implanted plastic stent with adequate position in the common bile duct and appropriate function.

Endoscopic retrograde ERCP was made and showed a stenosis of common bile duct 3 cm in length with prestenotic dilatation of both left and right hepatic duct and intrahepatic bile ducts (Fig. 4). A papilotomy was then made, brush aspiration for cytological analysis and a biliary plastic stent (Amsterdam 10F, 6 cm) was inserted at the site of stenosis with good bile drainage (Fig. 5).

**Figure 6:**
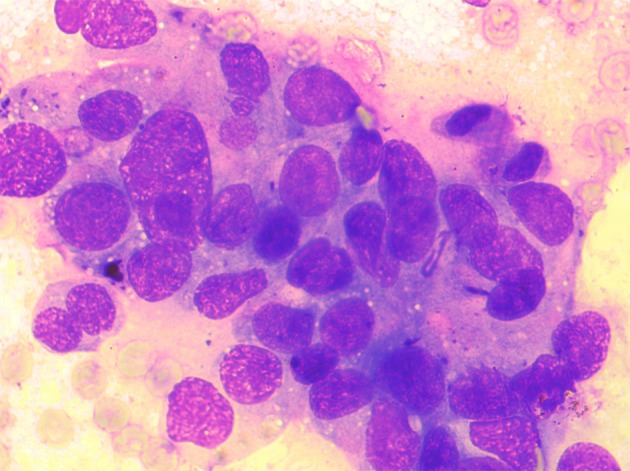
Cytological analysis of the material obtained during ERCP. The smears are showing several smaller and a few larger, often three-dimensional groups of partially degenerative, well-to-moderately differentiated malignant cells, originating from glandular epithelium (MGG, × 40).

Cytological analysis of the material obtained during ERCP showed several smaller and a few larger, often three-dimensional groups of partially degenerative, well-to-moderately differentiated malignant cells, originating from glandular epithelium. Morphological image does not meet the cytological criteria for cholangiocarcinoma, but suggests good-to-moderately differentiated adenocarcinoma (Fig. 6).

Three months after the plastic stent implantation we have replaced it with metal stent because of its better efficiency and lower complication rates. During patient’s 6 months follow-up we observed full ultrasonic and biochemical regression of cholestasis, the oncologist ordered aromatase inhibitors and our patient is at the moment without major subjective symptoms.

## Discussion

Breast cancer is the most commonly diagnosed cancer worldwide, with more than 1,384,000 cases detected in 2008 [1]. The incidence rates are the lowest in Asia and Sub-Saharan Africa, and the highest in North America, Australia, Western and Northern Europe [6]. Croatia is, according to Globocan estimates for 2008, among European countries with intermediate breast cancer incidence and mortality. Epidemiological studies showed that in Croatia, from 1988 to 2008, the number of new breast cancer cases increased from 1,220 to 2,472 with the number of death cases increasing from 670 to 902, which is comparable to trends in other European countries, particularly in Central and Eastern Europe [7].

Factors which affect prognosis of the disease include the size and grade of primary lesion, the presence of hormonal receptors, regional lymphadenopathy and distant metastatic disease [8]. Hematogenous metastases to lung, bone, liver and brain represent the most frequent sites of disease recurrence, while the involvement of gastrointestinal tract is rare and can pose a diagnostic challenge [9, 10].

Jaundice, as a symptom in patients with breast cancer, usually arises from metastatic disease replacing liver parenchyma, but there is also a group of patients whose jaundice is caused by obstruction of extrahepatic bile ducts by nodal metastases [11]. It is important to recognize this group of patients because in patients with normal liver function, relief of biliary obstruction using surgical bypass or biliary stenting extends their survival to over 1 year [12], in comparison to those with liver metastases, whose mean survival is only about 1 month [13]. Biliary stenting is a commonly used procedure in treating patients with pancreaticobiliary malignancies, metastatic disease and external biliary compression by lymph nodes. It is used both as a bridge to surgery in patients with resectable disease and for palliation in those with biliary obstruction caused by inoperative disease. In the market there is a variety of stents available, which can be divided into two main groups: plastic and metal [14]. Plastic stents are less expensive and can be easily removed if needed, but they eventually develop occlusion so they often require replacement. Metal stents, on the other hand, extend the duration of efficiency, but they have significantly higher costs and may not be removable. The most common complications of biliary stent implantation include stent occlusion and migration, and less common are infections (cholangitis, cholecystitis, pancreatitis), perforation and bleeding [15]. A meta-analysis on 2,436 patients with unresectable malignant biliary obstruction compared surgical bypass with endoscopic metal or plastic stent implantation. Plastic stents had lower risk of complications when compared with surgical bypass, but higher risk for biliary obstruction. When comparing metal with plastic stent, metal stents had a lower risk of recurrent obstruction at 4 months, but they were not superior to plastic stent with regard to technical and therapeutic success, mortality rate or complications [16].

In our case, first we implanted a plastic stent to gain symptomatic relief, but according to the literature, metal stent would be preferable, so we replaced plastic stent with metal one 3 months after the first intervention.

In conclusion, we want to emphasize the importance of thorough monitoring in patients with history of breast cancer, with particular accent on unusual symptoms and manifestations, which can be first signs of advanced disease. Although gastrointestinal involvement in breast cancer is rare, in the differential diagnosis of jaundice in a patient with a history of breast cancer, physicians should always consider the possibility of extrahepatic biliary metastasis, without liver involvement, especially because this condition is amenable to palliation and represents higher survival rate.
